# The Antioxidative Effect of Electro-Acupuncture in a Mouse Model of Parkinson's Disease

**DOI:** 10.1371/journal.pone.0019790

**Published:** 2011-05-23

**Authors:** Haomin Wang, Yanli Pan, Bing Xue, Xinhong Wang, Feng Zhao, Jun Jia, Xibin Liang, Xiaomin Wang

**Affiliations:** 1 Neuroscience Research Institute, Peking University, Beijing, People's Republic of China; 2 Science and Education Office, Beijing An Ding Hospital, Beijing, People's Republic of China; 3 Medical Experiment and Test Center, Capital Medical University, Beijing, People's Republic of China; 4 School of Public Health and Family Medicine, Capital Medical University, Beijing, People's Republic of China; 5 Department of Physiology, Capital Medical University, Key Laboratory for Neurodegenerative Disorders of the Ministry of Education, Beijing, People's Republic of China; 6 Department of Neurology and Neurological Sciences, Stanford University, Stanford, California, United States of America; National Institute of Health, United States of America

## Abstract

Accumulating evidence indicates that oxidative stress plays a critical role in Parkinson's disease (PD). Our previous work has shown that 100 Hz electro-acupuncture (EA) stimulation at ZUSANLI (ST36) and SANYINJIAO (SP6) protects neurons in the substantia nigra pars compacta from 1-methyl-4-phenyl-1,2,3,6-tetrahydropyridine (MPTP) toxicity in male C57BL/6 mice, a model of PD. In the present study we administered 100 Hz EA stimulation at the two acupoints to MPTP-lesioned mice for 12 sessions starting from the day prior to the first MPTP injection. We found that in the striatum of MPTP treated mice 100 Hz EA stimulation effectively inhibited the production of hydrogen peroxide and malonaldehyde, and increased glutathione concentration and total superoxide dismutase activity through biochemical methods. However, it decreased glutathione peroxidase activity via biochemical analysis and did not affect the level of 1-methyl-4-phenylpyridinium in the striatum revealed by high performance liquid chromatography with ultraviolet detection. These data suggest that 100 Hz EA stimulation at ST36 and SP6 has antioxidative effects in the MPTP model of PD. This data, along with our previous work, indicates that 100 Hz EA stimulation at ST36 and SP6 protects the nigrostriatal system by multiple mechanisms including antioxidation and antiapoptosis, and suggests that EA stimulation is a promising therapy for treating PD.

## Introduction

Parkinson's disease (PD) is a common neurodegenerative disease characterized by motor disorders resulting from the profound loss of dopaminergic neurons in the substantia nigra pars compacta (SNpc) and the subsequent depletion of dopamine (DA) in the striatum. Though significant progress has been made in the treatment of PD, no therapy has been proven to halt or slow disease progression or provide long-term improvement. Numerous investigations have focused on decoding the pathogenesis of PD in an attempt to find a therapeutic strategy. Several postmortem studies show that markers for lipid peroxidation, oxidative DNA and protein damage are significantly increased in the substantia nigra (SN) of PD patients [1]–[5], indicating that oxidative stress plays an important role in the pathogenesis of PD [6].

Administration of the toxin 1-methyl-4-phenyl-1,2,3,6-tetrahydropyridine (MPTP) causes neurochemical, behavioral, and histopathological alterations in human and nonhuman primates that replicate very closely the clinical symptoms of PD patients, so MPTP is widely used to produce animal models of PD [7]. MPTP is highly lipophilic and crosses the blood-brain barrier soon after systemic administration. In the brain MPTP is metabolized to 1-methyl-4-phenylpyridinium (MPP^+^), the active toxic compound. The formation and toxic production process of MPP^+^ are accompanied by an increased production of free radicals, especially superoxide [8], [9], which is poorly reactive but can be turned into hydrogen peroxide (H_2_O_2_). H_2_O_2_ participates in MPTP injury through forming hydroxyl radicals [10], the potent oxidants that attack DNA, protein and membrane lipids leading to cell death. Previous studies have suggested that antioxidants could protect DA neurons in the SNpc from MPTP injury [11]–[13], indicating that antioxidant therapy might be a potential therapeutic choice for PD.

Accumulating clinical evidences have demonstrated that acupuncture helps to improve movement disabilities and reduce the dosage of drugs required by PD patients [14]–[16]. ZUSANLI (ST36) and SANYINJIAO (SP6) are often used by acupuncturists to treat PD patients at their clinics on the basis of ancient theories of Traditional Chinese Medicine. Modern science research had shown that stimulation in these two acupoints could enhance the immunity and improve the mobility [17]–[20]. However, the underlying mechanisms are still unclear.

In this study, we hypothesized that the acupuncture stimulation has neuroprotective effect on DA neurons and this effect is stimulation frequency-dependent and is related to the antioxidative effect of acupuncture. We tested this hypothesis by evaluating the DA neuron quantity, the oxidative damage and levels of antioxidants after different frequency EA stimulation at ST36 and SP6 in MPTP treated mice.

## Materials and Methods

### Ethics statement

All animal experiments were performed by Haomin Wang, whose permit number of License for Performing Animal Experiments of Beijing, which is approved and required by the Ethics Committee of Peking University Health Science Center (a branch committee of the Committee on Animal Care and Usage of Peking University Health Science) before conducting animal experiments in Peking University Health Science Center, is 12928.

### Animals

Male C57BL/6 mice weighing 22∼25 g were supplied by the Laboratory Animal Center of Peking University, and housed in a temperature-controlled room (23±1°C) under 12-h on/off light cycle with food and water *ad libitum* in the home cage. Mice were allowed to acclimate to their home environment for 7 days before experiments.

### EA stimulation

**Figure 1:**
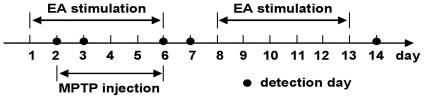
Experimental design of the study. Numbers represent days.

Mice were randomly divided into five groups: saline (NS), saline plus EA stimulation at 100 Hz (100 Hz + NS), MPTP, MPTP plus EA stimulation at 0 Hz or 100 Hz (0 Hz + MPTP and 100 Hz + MPTP respectively). The EA stimulation was performed from day 1 to day 13 except day 7 (Figure 1) as described before [21] with minor modifications. The mouse was gently restrained in a polyethylene cylinder with its hind limbs and tail outside. Two sterilized stainless-steel needles 0.18 mm in diameter and 3 mm long were inserted in each leg, one at ST36 (2 mm lateral to the anterior tubercle of tibia) and the other at SP6 (2 mm proximal to the upper border of medial melleolus, at the posterior border of the tibia). Bidirectional square wave electrical pulses (0.2 ms duration, 100 Hz) or no electrical pluses (0 Hz), designated as EA, were given for a total of 30 min each day. The intensity of the stimulation at 100 Hz was increased stepwise from 1 to 1.25 mA and then to 1.5 mA, with each step lasting for 10 min. The animals remained relaxed during stimulation, so anesthesia was not performed.

### MPTP treatments

Following EA stimulation mice received intraperitoneal (i.p.) injections of MPTP from day 2 to day 6 (Figure 1) (Sigma-Aldrich, St. Louis, MO, USA, 30 mg/kg, dissolved in saline, once a day) or an equivalent volume of saline.

### Tissue collection and processing

Three mice from each group were randomly selected on day 14 for tyrosine hydroxylase (TH) immunohistochemistry. They were deeply anesthetized with 400 mg/kg chloral hydrate, and then transcardially perfused with 25 ml saline followed by 75 ml 4% (w/v) paraformaldehyde in phosphate buffer. Brains were removed and post-fixed in the same fixative overnight and then cryoprotected in 30% (w/v) sucrose for 3 ∼ 5 days. The brains were frozen on powdered dry ice and then arranged for frontal sectioning according to the mouse brain atlas of Burton M. Slotnick and Christina M. Leonard. Brains were sectioned at 20 µm thickness with a cryostat at −20°C and processed for immunohistochemistry. On day 2 (2 hr. post MPTP injection), 3 (4 hr. post MPTP injection), 6 (2 hr. post MPTP injection), 7 and 14, seven to eight mice from each group were decapitated, and the bilateral striata and the ventral midbrains were dissected quickly and stored at −80°C (Figure 1).

### Immunohistochemistry and quantification of TH-ir neuronal profiles

All sections spanning the SN were collected for immunohistochemistry according to the previously described method [22] with minor modifications. Every seventh section was incubated in rabbit anti-TH antibody (1∶2000, Chemicon, Temecula, CA, USA) at 4°C overnight. Sections treated with diluted non-immune goat serum instead of primary antibody served as an antibody control. Sections were incubated with biotinylated goat anti-rabbit antibody and then with the avidin–biotin–peroxidase complex for 30 min at 37 °C. The bound complex was visualized by incubating sections in a solution containing 0.1% (w/v) 3,3-diaminobenzidine (Sigma, St. Louis, MO, USA), 1% (v/v) H_2_O_2_, and 8% (w/v) ammonium nickel sulfate (Fluka Chemie GmbH, Switzerland).

TH-ir neuronal profiles with distinct nuclei were counted in ten sections throughout the entire rostrocaudal extent of the SNpc. All sections were coded and examined blind.

### HPLC analysis of dopamine and its metabolites

Striata collected on day 14 were used to detect the levels of DA and its metabolites, dihydroxyphenylacetic acid (DOPAC) and homovanillic acid (HVA), by HPLC with electrochemical detection (HPLC-ECD). In brief, tissues were weighed and then homogenized in 0.4 M ice-cold perchloric acid (150 µl/tissue). All homogenates were kept away from light in an ice bath for 60 min. Centrifuging at 12,000 rpm and 4°C for 20 min, transferring 120 µl supernatant from each sample to a new tube and then adding 60 µl solution (20 mM potassium citrate, 300 mM potassium dihydrogen phosphate, and 2 mM EDTA-2Na). Keeping the mixtures away from light in an ice bath for 60 min, and then centrifuging at 12,000 rpm and 4°C for 20 min. Filtering the supernatant with a 0.22 µm Millipore filter and injecting the filtrate into the HPLC system for analysis. The mobile phase contained 110 mM citrate buffer/100 mM EDTA/70 mM 1-octanesulfonate sodium solution and 20% (v/v) methanol. Flow rate was 1 ml/min. Striata from six to nine animals in each group were used.

### H_2_O_2_, MDA, total SOD, GSH, and GSH-PX assay

On day 3, 7 and 14 mice were sacrificed and the striata as well as the ventral midbrains were dissected as described above. About seven striata and ventral midbrains from each group were homogenized in 30 vol. (wt./vol.) of 0.1 M phosphate buffer solution and centrifuged at 3000 g and 4°C for 15 min. The supernatant was used to determine the level of H_2_O_2_, malonaldehyde (MDA) and activity of total superoxide dismutase (SOD). The supernatant from the striata and the ventral midbrains diluted in 10 vol. (wt./vol.) buffer was used for glutathione peroxidase (GSH-PX) activity assay. H_2_O_2_, MDA, SOD and GSH-PX assays were performed according to the procedures provided by the assay kits (Nanjing Jiancheng Bioengineering Institute, Nanjing, PR China). The glutathione (GSH) content was detected by a Total Glutathione Quantification Kit (Dojindo Laboratories, Kumamoto, Japan), following the kits' instructions. H_2_O_2_ content was determined by monitoring at the absorbance at 412 nm of the titanium-peroxide complex [23]. MDA level was analyzed with 2-thiobarbituric acid [24]. SOD activity was analyzed by monitoring the inhibition of the reduction of nitro blue tetrazolium by the sample at 550 nm [25]. GSH-PX activity was detected with 5-5′-dithiobis-p-nitrobenzoic acid [26]. GSH level was measured by DTNB-GSSG reductase recycling assay [27]. All the assays were colorimetric methods based on biochemical reactions, and the absorbance values of the samples were calibrated against that of the standards with known concentration or calibrated to a standard graph generated with known content of the standards.

### MPP^+^ measurement

Striata collected on day 2 and 6 (2 hr. after the first and last injection of MPTP) were used for measuring MPP^+^ level using HPLC with UV detection (HPLC-UV, wavelength, 293 nm). Samples were weighed, homogenized in 200 µl ice-cold perchloric acid (0.1 M). and then centrifuged at 12,000 rpm at 4°C for 7 min. The supernatant was filtered prior to analysis by HPLC. For HPLC analysis the mobile phase contained 85% (v/v) 0.1 M acetic acid/75 mM triethylamine solution and 15% (v/v) acetonitrile and the flow rate was 1 ml/min.

### Statistical analysis

Values are expressed as mean ± SEM. Differences among means were analyzed using one-way ANOVA followed by Newman–Keuls post hoc test of difference between means. A *p* value <0.05 denoted a statistically significant difference.

## Results

### 100 Hz EA stimulation protects dopaminergic neurons from MPTP toxicity

**Figure 2:**
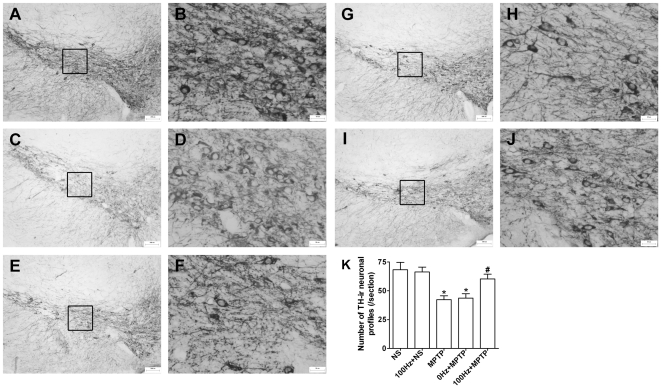
100 Hz EA stimulation protects dopaminergic neurons from MPTP toxicity. (A and B) NS. (C and D) 100 Hz + NS. (E and F) MPTP. (G and H) 0 Hz + MPTP. (I and J) 100 Hz + MPTP. (K) Quantification of TH positive neuronal profiles in the SNpc. *p<0.05, compared with NS group. n = 3. Scale bar, 200 µm (A, C, E, G and I) and 50 µm (B, D, F, H and J).

Profound loss of DA neurons in the SNpc is the main pathological change of PD. Here we assessed whether EA stimulation could rescue DA neurons in the SNpc from MPTP toxicity by TH immunohistochemistry. We found that on day 14, TH positive neurons in MPTP treated mice dramatically decreased (*p*<0.05 *vs.* NS group; Figure 2, E and F), in comparison with the saline group (Figure 2, A and B). However, TH immunoreactivity could be rescued by 100 Hz EA stimulation (*p*<0.05 *vs.* MPTP group; Figure 2, I and J). Unlike 100 Hz, EA stimulation at 0 Hz made no difference (Figure 2, G and H). Furthermore, 100 Hz EA stimulation had no effect on saline treated control mice (Figure 2, C and D). These results suggest that 100 Hz EA stimulation can protect DA neurons in the SNpc from MPTP injury.

### 100 Hz EA stimulation increases the concentration of striatal DA and its metabolites in PD mice

**Figure 3:**
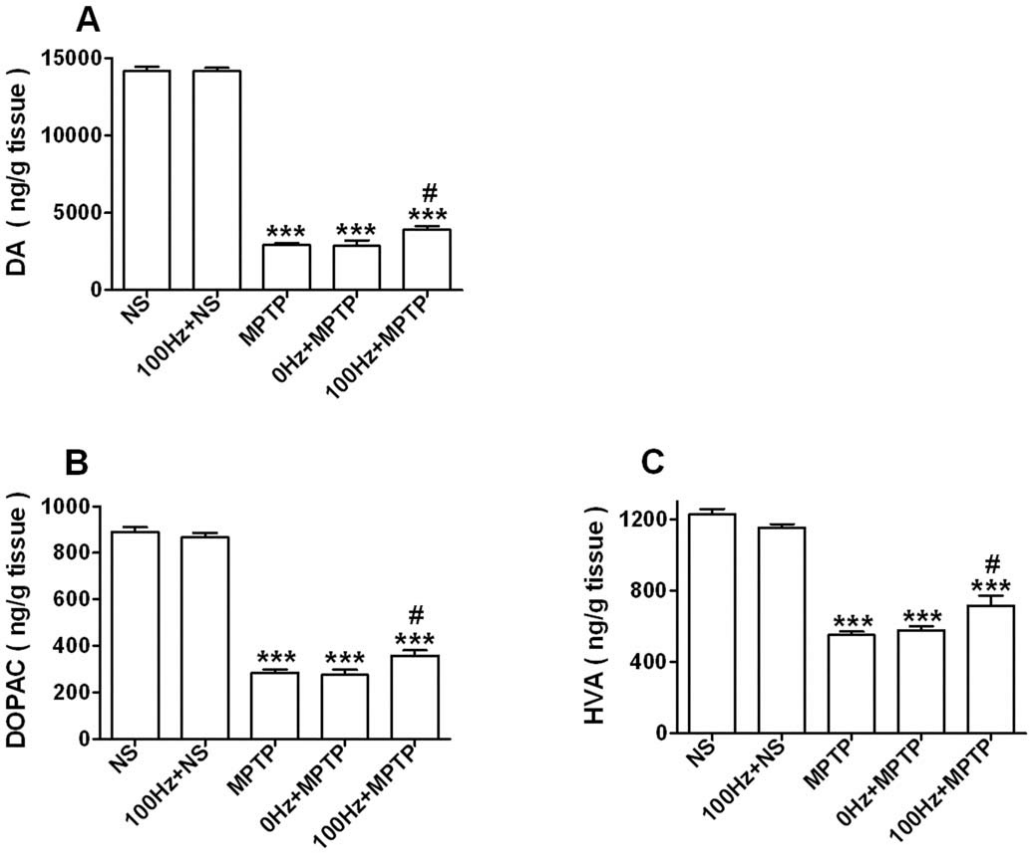
100 Hz EA stimulation increases the contents of striatal DA and its metabolites in MPTP-treated mice. (A) DA. (B) DOPAC. (C) HVA. ***p<0.001, compared with NS group; #*p*<0.05, compared with MPTP group. n = 6∼9.

Because the abnormal motor function in PD is mainly caused by subthreshold levels of DA in the striatum, we looked at the concentration of striatal DA and its metabolites, DOPAC and HVA in our different groups. On day 14, MPTP injection caused a significant reduction in the concentration of the three substances (*p*<0.001 *vs.* NS group, Figure 3). However, 100 Hz EA stimulation elevated DA levels significantly (34% increase, *p*<0.05 *vs.* MPTP group, Figure 3A) in the MPTP treated mice, as well as DOPAC and HVA concentrations (19.8% and 22.9% increase respectively, *p*<0.05 *vs.* MPTP group; Figure 3, B and C). Consistent with the TH immunohistochemistry results, 0 Hz EA stimulation did not affect the concentrations of DA, DOPAC and HVA in the striatum of the MPTP treated mice.

### 100 Hz EA stimulation inhibits the elevation of striatal H_2_O_2_ level in PD model mice

**Figure 4:**
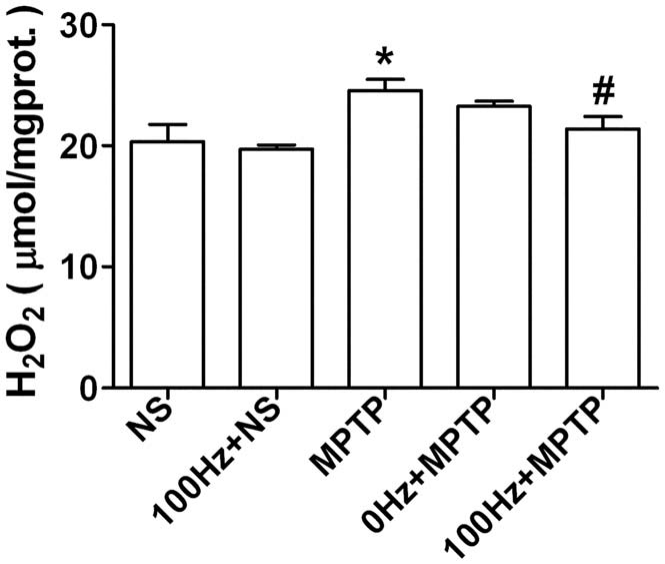
100 Hz EA stimulation inhibits the elevation of striatal H_2_O_2_ level in MPTP-treated mice. *p<0.05, compared with NS group; #*p*<0.05, compared with MPTP group. n = 5∼8.

In our model, striatal H_2_O_2_ content increased significantly at 2 hr. after a single MPTP injection and reached its peak at 4 hr. (Figure S1). Measurement of striatal H_2_O_2_ level at 4 hr. after every MPTP injection (five injections in total) show that only the first three MPTP injections augment H_2_O_2_ levels significantly (Figure S1 and S2). Therefore we examined striatal H_2_O_2_ level on day 3 when mice had been given two MPTP injections. The results show that 100 Hz EA stimulation inhibits the elevation of H_2_O_2_ in MPTP treated mice (*p*<0.05 *vs.* MPTP group, Figure 4), while, 0 Hz EA stimulation has no affects. Additionally, 100 Hz EA stimulation had no effect on normal mice. Moreover, we observed there was no significant change of H_2_O_2_ contents in the ventral midbrain of the model mice compared with the NS group (Figure S3 and S4).

Since all of our above tests show that 100 Hz EA stimulation had no adverse effect on normal mice and 0 Hz EA stimulation did not have an effect on MPTP treated mice, we abandoned the 100 Hz + NS and 0 Hz + MPTP groups in order to minimize the number of animals used in the following experiments.

### Effects of 100 Hz EA stimulation on the concentration/activity of striatal GSH, GSH-PX and SOD

In the brain, major antioxidant defenses consist of antioxidant scavengers such as GSH and enzymes such as GSH-PX and SOD. For the following experiment we measured striatal concentration and activity of GSH, GSH-PX and total SOD on day 3, 7 and 14.

**Figure 5:**
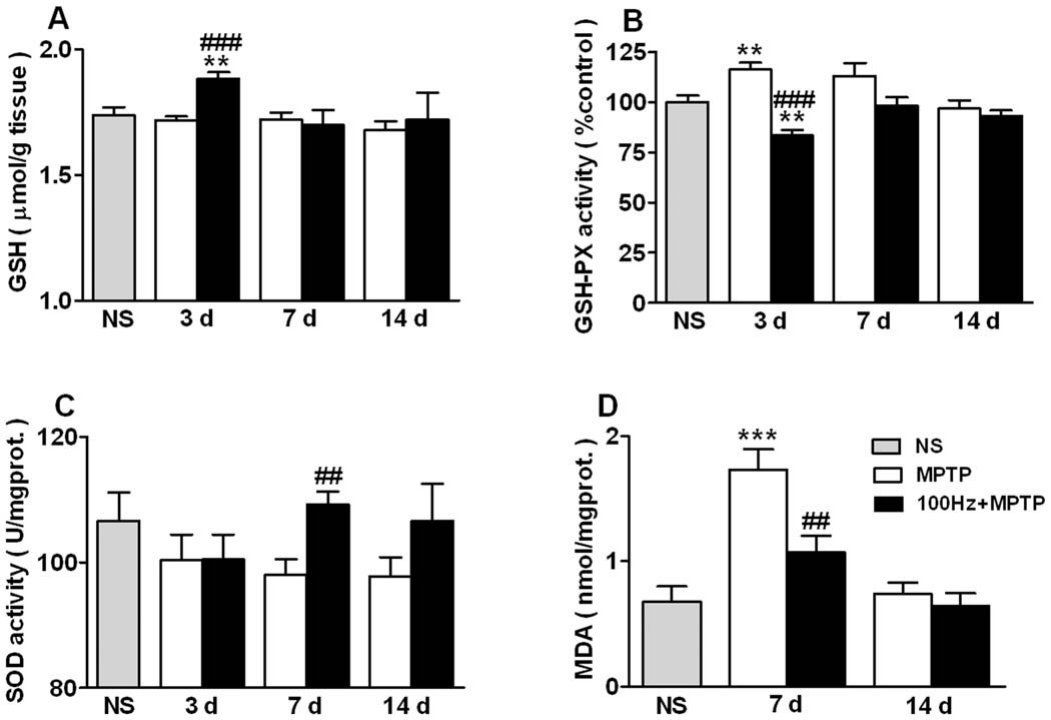
100 Hz EA stimulation effects on the content/activity of GSH, GSH-PX, SOD and MDA in the striatum. Saline group (gray bar), MPTP group (white bar) and 100 Hz + MPTP group (black bar). (A) GSH content. (B) GSH-PX activity. (C) SOD activity. (D) MDA content. **p<0.01, ***p<0.001, compared with NS group; ##*p*<0.01, ###*p*<0.001, compared with MPTP group on the same day. n = 6∼7 (A and B) or n = 5∼7 (C and D).

On day 3 EA stimulation enhanced GSH content significantly (*p*<0.001 *vs.* NS and *p*<0.001 *vs.* MPTP group on day 3, Figure 5A), but the effect disappeared on day 7 and 14. MPTP injection did not affect GSH content in the striatum.

GSH-PX activity was significantly increased on day 3 in the MPTP group (*p*<0.01 *vs*. NS, Figure 5B). 100 Hz EA stimulation significantly decreased GSH-PX activity at that time point (*p*<0.01 *vs*. NS group). On day 7, high levels of GSH-PX activity were still seen in the MPTP treated mice (13.3% increase compared to NS group, Figure 5B) but EA stimulation normalized GSH-PX activity in model mice. On day 14, MPTP and EA stimulation had no effect on GSH-PX activity.

SOD activity was decreased in the striatum of MPTP treated mice on all of the three time points, i.e., day 3, day 7 and day 14 (6.0% ∼ 8.3% compared to NS group, Figure 5C). 100 Hz EA stimulation increased the SOD activity in a time dependent manner, i.e., 3 sessions (day 3) of treatment did not affect SOD activity, 6 sessions (day 7) significantly increased SOD activity (8.8% increase compared to EA group on day 3, *p*<0.01 *vs*. MPTP group on day 7, Figure 5C) and 12 sessions (day 14) of treatment also increased SOD activity too.

### 100 Hz EA stimulation depresses the elevation of striatal MDA content

MDA is one of the final products of polyunsaturated fatty acid peroxidation in cells. An increase in free radicals causes overproduction of MDA. Therefore, it is used as a lipid peroxidation marker. We detected striatal MDA content on day 7 and 14, the 1^st^ and 8^th^ day after the last MPTP injection respectively. On day 7, MDA levels were significantly increased in MPTP treated mice (155% increase, *p*<0.001 *vs*. NS group, Figure 5D) but EA stimulation reduced this increase (38% decrease, *p*<0.01 *vs*. MPTP group on day 7). On day 14 there were no statistic differences among the three groups. Moreover, we found there was no significant change of MDA levels in the ventral midbrain of the model mice compared with the NS group (Figure S5).

### 100 Hz EA stimulation does not affect MPP^+^ metabolism

**Figure 6:**
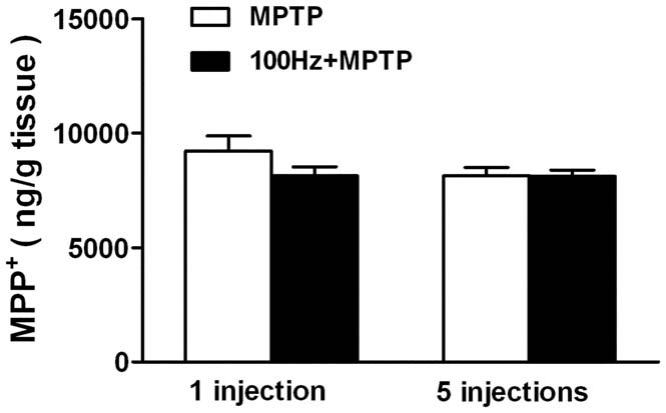
100 Hz EA stimulation does not affect MPP^+^ formation. MPTP group (white bar) and 100 Hz + MPTP group (black bar). n = 6∼8.

In the brain the toxicity of MPTP is due to its toxic form, MPP^+^, which is selectively toxic to dopaminergic neurons. We evaluated if the antioxidative effect of EA stimulation was related to the formation or degradation of MPP^+^. On day 2 and day 6 when the 1^st^ and 5^th^ MPTP injections were performed, mice were killed for the analysis of striatal MPP^+^ content by HPLC-UV. Our data shows that EA stimulation does not influence the concentration of MPP^+^ in the striatum of the MPTP treated mice (Figure 6), suggesting that the target of EA stimulation at 100 Hz does not involve in the MPP^+^ metabolic pathway.

## Discussion

More and more people turn to acupuncture for the treatment of Parkinson's disease and clinical evidence has proven the effectiveness of acupuncture in the management of this dread disease. But the underlying mechanism still needs to be clarified. In this study we found that 100 Hz, but not 0 Hz of EA stimulation at ST36 and SP6 can protect dopaminergic neurons in the substantia nigra from MPTP insult, suggesting that the response of the body to EA stimulation is frequency-dependent. Although multiple mechanisms may be involved in this process, our findings highlight the possibility that the antioxidative effect of EA stimulation may be a leading mechanism. Oxidative stress is involved in dopaminergic neuronal injury in MPTP-lesioned mice. EA at 100 Hz reverses the elevation of striatal MDA concentration in PD model mice. This antioxidative activity of EA partially relies on its ability to reduce H_2_O_2_ content and elevate GSH level and total SOD activity. This activity also depends on frequency because 0 Hz EA stimulation did not benefit PD mice. In addition, 100 Hz EA stimulation did not adversely affect normal mice.

In tissues obtained at autopsy from PD patients the activity of SOD is increased, while GSH-PX activity and GSH content are decreased [28]–[30]. SOD is often regarded as the first line of defense against an upswing of reactive oxygen species (ROS) and responsible for the conversion of superoxide to H_2_O_2_ in the cytoplasm and mitochondria. Enhanced SOD activity may be neuroprotective since transgenic mice with increased SOD activity are resistant to MPTP injury [31], [32], while mice with decreased SOD activity are more susceptible to MPTP toxicity [10], [33]. GSH is considered to be a major antioxidant in the brain, capable of attenuating oxidative damage [34]. Impairment of the GSH system may trigger a cascade of events leading to oxidative stress and destruction of the nigrostriatal pathway as well as render the pathway susceptible to a toxic insult [35]. GSH depletion is a primary event in incidental Lewy body disease which is thought to be presymptomatic Parkinson's disease. GSH-PX is an enzyme of major importance in the detoxification of peroxides such as H_2_O_2_. Deficiency of GSH-PX activity leads to aggravating MPTP lesions [10]. Ebselen, an antioxidant drug with GSH-PX-like activity, prevents both neuronal loss and clinical symptoms in a primate MPTP model of PD [13].

Our findings reveal that 100 Hz EA stimulation at ST36 and SP6 can prevent the decrease of striatal total SOD activity, elevate striatal GSH concentration, and consequently inhibit the increase of striatal H_2_O_2_ and MDA level caused by MPTP. On day 3 (3 sessions of EA) the decrease of striatal GSH-PX activity in the EA group might relate to the augmented striatal GSH content, which helps to consume the excessive H_2_O_2_.

Recently, Yu et al. claimed that acupuncture mitigated oxidative stress in the SN of 6-hydroxydopamine lesioned rats [36]. Compared with their study we used MPTP mice model, which is the best available and the most popular animal model of PD at present [7], [9], [37]–[41]. Furthermore, we detected the oxidative indicators in a time-course manner (on day 3, 7 and 14), and illustrated a picture on the oxidative changes in MPTP mice model. In our model the rapid elevation of H_2_O_2_ content and GSH-PX activity suggests that the production of ROS is an early event in MPTP toxicity, consistent with the observations in other experiments [42], [43]. Also, our study suggested that oxidative stress could be more profound in the striatum than that in the ventral midbrain, which might be due to the fact that the DA neuron loss induced by MPTP results from molecular events initiated in the striatum [44]–[46]. Thus, the antioxidative effect of EA at these two acupoints on the striatum could be significant to rescue the DA neurons in the SN. Kim et al. found that 100 Hz EA normalized the elevation of glyoxalase II, which plays a pro-survival role in the metabolic stress response through detoxifying methylglyoxal in MPTP mice, and they assumed that it could be due to the relief of oxidative stress in the striatum by increasing antioxidant enzyme activities, thereby precluding methylglyoxal accumulation [47].

Motor behavioral abnormality is the cardinal characteristics of human PD. Therefore, therapies that can improve the abnormal behavior will significantly help PD patients in their daily life. In this study we found that 100 Hz EA stimulation normalized the motor disorders of the model mice. We think that the mechanism is due to the regulatory effect of EA on other nuclei in the basal ganglia, such as the globus pallidus, but not the neuroprotective effect of EA on the dopaminergic neurons in the nigrostriatal system (Wang HM et al. unpublished). It is in accordance with the previous studies in our lab [48]–[50].

MPP^+^ activates microglia which exaggerates its toxicity via ROS dependent and independent mechanisms [51]. Our previous work revealed that 100 Hz EA stimulation can suppress the activation of microglia and up-regulate BDNF and GDNF expression in medial forebrain bundle-transected PD rats [22], [50], [52]. Therefore, 100 Hz EA stimulation might rescue DA neurons through multiple ways besides mitigating oxidative stress in MPTP mice. Indeed, we have discovered that 100 Hz EA stimulation at ST36 and SP6 has an anti-apoptotic effect by elevating the Bcl-2/Bax ratio in this model (Pan YL et al., unpublished).

In its late stage PD destroys multiple regions of the brain except for the nigrostriatal system, which leads to complex clinical symptoms such as pain and insomnia. A clinical report demonstrated that acupuncture benefited the sleep of PD patients and eased the patients’ subjective sufferings from pain [53] suggesting that acupuncture stimulation produces extensive neuroprotective and regulative effects. Therefore, it is highly possible that the integration of several activated signal pathways during acupuncture stimulation plays a role in alleviating the pathological changes in the brain of PD patients.

## Supporting Information

Figure S1 **Time course of striatal H_2_O_2_ levels after a single injection of MPTP.** ** p<0.01, ***p<0.001, compared with NS group. n = 5∼7.
(TIF)

Figure S2 **Time course of H_2_O_2_ contents in the striatum of the subacute MPTP mouse model.** *p<0.05, **p<0.01, compared with NS group. n = 6.
(TIF)

Figure S3 **Time course of H_2_O_2_ levels in the ventral midbrain after a single injection of MPTP.** Animals were sacrificed at 2, 4, 6, 8, 10 and 12 hours post one MPTP injection (30 mg/kg, i.p.). H_2_O_2_ contents of the ventral midbrains were detected. n = 5∼7.
(TIF)

Figure S4 **Time course of H_2_O_2_ contents in the ventral midbrain of the subacute MPTP mouse model.** At 4 hours after the 2^nd^, 3^rd^, 4^th^ and 5^th^ MPTP injection (30 mg/kg, i.p.), animals were decapitated. Contents of H_2_O_2_ in the ventral midbrains were detected. n = 6.
(TIF)

Figure S5 **Time course of MDA contents in the ventral midbrain of the subacute MPTP mouse model.** After the 2^nd^, 3^rd^, 4^th^ and 5^th^ MPTP injection (30 mg/kg, i.p.), animals were decapitated. Contents of MDA in the ventral midbrains were detected. n = 6.
(TIF)
